# Glioma-derived plasminogen activator inhibitor-1 (PAI-1) regulates the recruitment of LRP1 positive mast cells

**DOI:** 10.18632/oncotarget.4640

**Published:** 2015-06-25

**Authors:** Ananya Roy, Antoine Coum, Voichita D. Marinescu, Jelena Põlajeva, Anja Smits, Sven Nelander, Lene Uhrbom, Bengt Westermark, Karin Forsberg-Nilsson, Fredrik Pontén, Elena Tchougounova

**Affiliations:** ^1^ Uppsala University, Department of Immunology, Genetics and Pathology, Rudbeck Laboratory, Uppsala, Sweden; ^2^ Nanoscience Centre, Department of Engineering, Cambridge University, Cambridge, UK; ^3^ Uppsala University, Department of Immunology, Genetics and Pathology, and Science for Life Laboratory, Rudbeck Laboratory, Uppsala, Sweden; ^4^ Cancer Research Technology, LBIC, London, UK; ^5^ Uppsala University, Department of Neuroscience, Neurology, Uppsala, Sweden; ^6^ Present address: Danish Epilepsy Center, Dianalund, Denmark

**Keywords:** mast cell, glioma, PAI-1, SERPINE1, LRP1

## Abstract

Glioblastoma (GBM) is a high-grade glioma with a complex microenvironment, including various inflammatory cells and mast cells (MCs) as one of them. Previously we had identified glioma grade-dependent MC recruitment. In the present study we investigated the role of plasminogen activator inhibitor 1 (PAI-1) in MC recruitment.

PAI-1, a primary regulator in the fibrinolytic cascade is capable of forming a complex with fibrinolytic system proteins together with low-density lipoprotein receptor-related protein 1 (LRP1). We found that neutralizing PAI-1 attenuated infiltration of MCs. To address the potential implication of LRP1 in this process, we used a LRP1 antagonist, receptor-associated protein (RAP), and demonstrated the attenuation of MC migration. Moreover, a positive correlation between the number of MCs and the level of PAI-1 in a large cohort of human glioma samples was observed. Our study demonstrated the expression of LRP1 in human MC line LAD2 and in MCs in human high-grade glioma. The activation of potential PAI-1/LRP1 axis with purified PAI-1 promoted increased phosphorylation of STAT3 and subsequently exocytosis in MCs.

These findings indicate the influence of the PAI-1/LRP1 axis on the recruitment of MCs in glioma. The connection between high-grade glioma and MC infiltration could contribute to patient tailored therapy and improve patient stratification in future therapeutic trials.

## INTRODUCTION

The heterogeneity of high-grade gliomas in clinical presentation, tumor location, growth pattern, response to therapy and consequently patient outcome continues to pose a challenge for clinical management [1]. Currently, the treatment of glioblastoma (GBM) involves maximal safe surgery, followed by radiation and chemotherapy. There are also additional experimental treatments, such as passive and active immunotherapy, use of angiogenic inhibitors in combination with chemotherapeutics and gene/antibody therapy [2]. Despite such aggressive treatment, the prognosis for high-grade gliomas remains among the poorest, with a median survival for patients with GBM of just over one year [3].

The disruption of the blood brain barrier (BBB) as a result of abnormal neovasculature and redundant vessel leakiness is one of the important hallmarks of GBM [4]. Another significant hallmark of GBM is its invasiveness into surrounding brain parenchyma. During recent years, the impact of microenvironment in tumor development has become increasingly acknowledged [5]. Brain tumor-associated parenchymal cells such as vascular cells, peripheral immune cells including microglia, myeloid suppressor cells and T-regulatory cells play a vital role in controlling the development of pathological conditions. Glioma is capable of hiding signals important for immune activation and subsequently ameliorating the environment for immunosuppressive factors [6]. Addressing the complexity of the unique glioma-immune cell interactions represents a crucial aspect for the future therapy of glioma.

Mast cells (MCs) are key regulators of the tumor microenvironment, affecting angiogenic processes, immune modulation and tissue remodeling [7]. The role of MCs in cancer is still controversial depending on the type and malignancy grade of the cancer. We were first to report that gliomas also contain MCs and we addressed the role for different axes in chemotactic infiltration of MCs into glioma [8, 9].

Plasminogen activator inhibitor 1 (PAI-1) or SERPINE1, a primary regulator in the plasminogen-plasmin system, is known as the principal inhibitor of urokinase type plasminogen activators (uPA) and tissue type plasminogen activators (tPA) by which it inhibits plasminogen activation. It is also capable of forming a complex with uPA and the uPA receptor (uPAR). This complex can be removed from the cell surface by binding with LRP1, followed by lysosomal degradation of PA-PAI-1 complexes and reappearance of uPAR on the cell surface, adjusting locomotion and the direction of migration of cells [10]. Previous publications demonstrated that overexpression of PAI-1 in GBM is significantly correlated with shorter survival [11]. In addition, the PAI-1 serum level was shown to be a predictive marker of glioma grade [12].

Here, we demonstrate that accumulation of MCs correlates with the level of PAI-1 in glioma cells, and also in tissue microarrays (TMAs) of a large cohort of human high-grade glioma tissues. We show that PAI-1 neutralizing antibodies attenuated the infiltration of MCs. Furthermore, to address the potential implication of low-density lipoprotein receptor-related protein 1 (LRP1) in this process, we demonstrated the expression of LRP1 in LAD2 cells but also revealed the presence of LRP1 in MCs in human high-grade glioma tissue. To properly assess the role of LRP1 in MC migration, we used a LRP1 antagonist, receptor-associated protein (RAP), and demonstrated the attenuation of MC migration upon LRP1 blocking. In addition, the activation of potential PAI-1/LRP1 axis with purified PAI-1 promoted differential phosphorylation of a number of signaling molecules including significant upregulation of pSTAT3, which, in turn, mediated MC exocytosis.

In conclusion, the present investigation reveals a potential mechanism by which the recruitment of MCs to glioma is mediated by tumor derived PAI-1. Our findings implicate the interaction between PAI-1 and the newly identified MC receptor LRP1 in pSTAT3 dependent MC exocytosis.

## RESULTS

### High SERPINE1/PAI 1 expression in human glioma correlates with low survival rate

We have earlier shown [9] that glioma derived macrophage migration inhibitory factor (MIF) is a potent chemoattractant for MCs. Another mediator that was highly expressed by glioma cells is PAI-1/SERPINE1.

**Figure 1:**
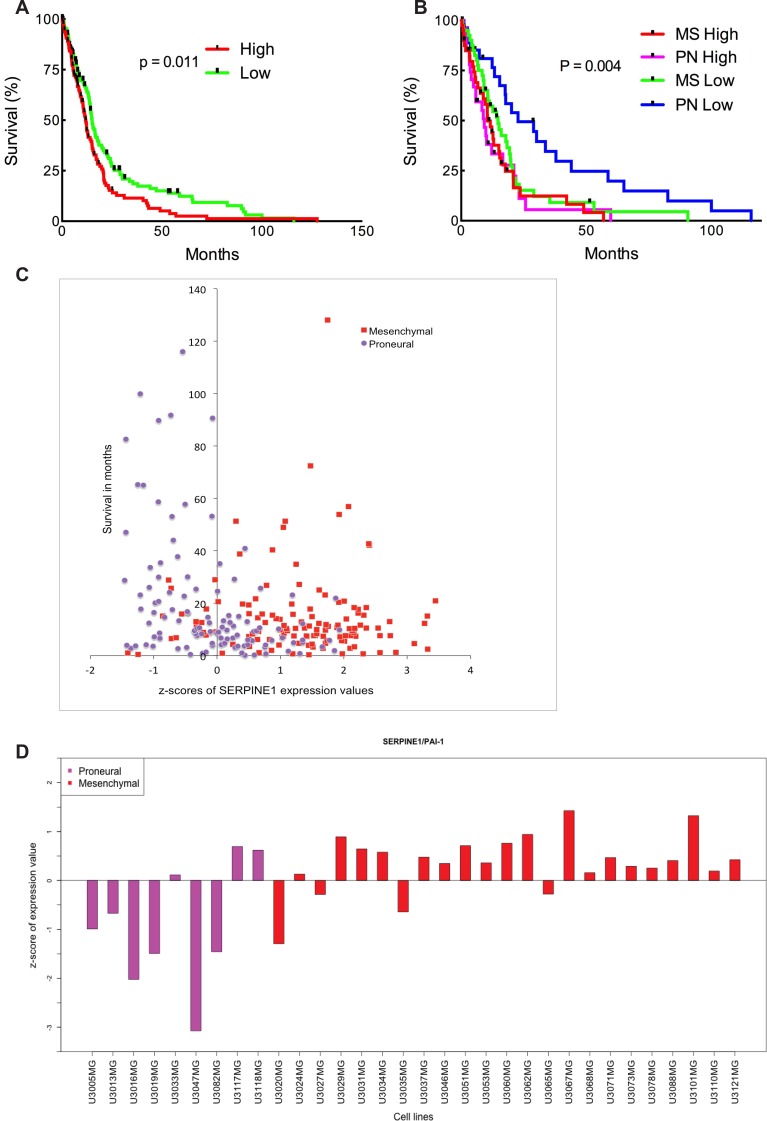
High SERPINE1 expression in human glioma correlates with low survival rate (**A**) Survival analysis comparing patient survival with high and low SERPINE1 expression. Log-rank *p*-value = 0.011 (n = 260). (**B**) Survival analysis of the samples of Mesenchymal (MS) high/low SERPINE1 (n = 80) and Proneural (PN) high/low SERPINE1 (n = 56) subtypes. Clinical data for the TCGA patients was obtained from the TCGA portal and in each case the maximum value of the survival time available was used. Significant differences in survival between groups were evaluated using a Kaplan-Meier analysis with censoring at confidence interval 95%. Log-rank *p*-value = 0.004. (**C**) z-scores of expression values for SERPINE1 versus survival in patients with Proneural and Mesenchymal glioma subtypes. Data obtained from the TCGA portal. (**D**) z-scores of expression values for SERPINE1 in HGCC cell lines of Proneural and Mesenchymal subtypes.

The recent classification of GBM has been given by The Cancer Genome Atlas (TCGA) network. Based on gene expression patterns for signature genes, genomic abnormalities and gene expression alterations in genes such as EGFR, NF1 and PDGFRA/IDH1, the TCGA GBM samples were classified in four subtypes, Proneural, Neural, Classical and Mesenchymal [13]. In order to assess the potential correlation between SERPINE1 signaling in human GBM we analyzed the TCGA dataset of 518 GBM patient samples and found a significant upregulation of SERPINE1 (data not shown). We used the well-established markers EGFR and PTEN as positive and negative controls for upregulated and downregulated expression in human GBM, respectively. To assess the correlation between SERPINE1 expression and patient survival we analyzed the TCGA GBM dataset consisting of 518 GBM patients for which transcriptome and survival data is available. First, we sorted the TCGA patients by SERPINE1 expression from high to low and performed a Kaplan-Meier survival analysis to compare the top 25% patients (with high expression) and the bottom 25% of patients (with low expression). This analysis showed a statistically significant difference in survival between the two groups (Figure 1A, log-rank *p*-value = 0.011). Further inspection of these groups showed that 83% of the patients with high SERPINE1 expression are of Mesenchymal subtype (MS) and 29% of the patients with low SERPINE1 values are of Proneural subtype (PN) (Figure 1C). To investigate this further, we selected, based on SERPINE1 expression (high or low), the top 25% and bottom 25% of patient samples within MS and PN subgroups, respectively, and performed a survival analysis between the resulting four groups. As shown in Figure 1B we found a statistically significant survival difference between the PN subgroup with low SERPINE1 expression (longer survival) and the other three (MS high/low SERPINE1 and PN high SERPINE1 expression).

Patient-derived glioma cell cultures used in this study are part of the Uppsala University Human Glioma Cell Culture (HGCC) collection that comprises well-characterized GBM-derived cell cultures (Xie et al 2015, submitted). A survival analysis based on the HGCC data was inconclusive owing to low sample numbers in comparison to the number of patients in the TCGA dataset. We chose cell lines from the HGCC database with high and low SERPINE1 expression (Figure 1D) from both Mesenchymal and Proneural subtypes to continue our investigation.

### Glioma derived PAI-1 promotes MC recruitment

**Figure 2:**
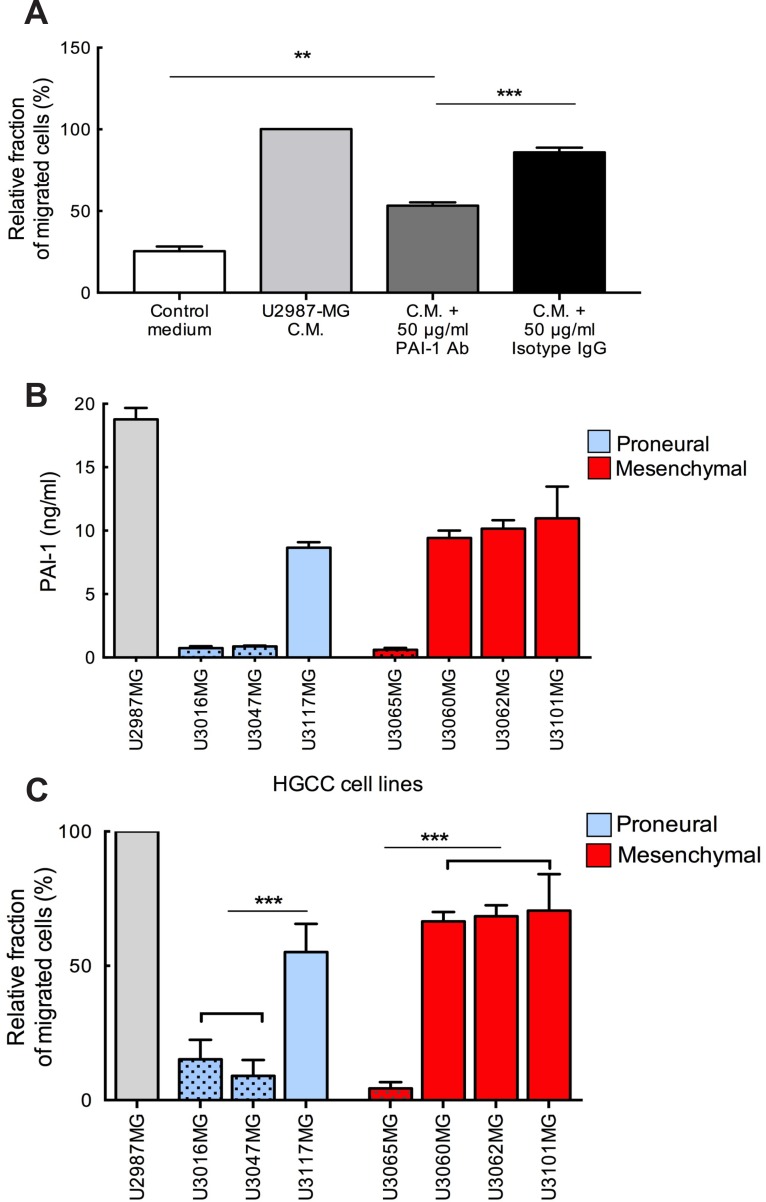
Neutralization of glioma-derived PAI-1 attenuates the migration of MCs toward conditioned medium (**A**) Migration of MCs towards conditioned media from U2987MG glioma cell line. The experiments were performed 3 times, with duplicates in each case. The error bars represent the SD. ** *p* < 0.01, *** *p* < 0.001. (**B**) PAI-1 levels in the media of U2987MG cell line grown in serum containing medium, in 3 Proneural and 4 Mesenchymal glioma cell line obtained from the HGCC collection and grown in neurobasal media. (**C**) Migration of MCs towards conditioned media from cultures of glioma cells from HGCC with high or low PAI-1 expression. C.M. = conditioned medium. Three independent experiments with triplicates were performed. The error bars represent the SD. *** *p* < 0.001.

Previously we demonstrated that human glioma cells secrete a number of mediators among which MIF promotes human MC recruitment [9]. Here we investigated the role of another candidate, PAI-1, which is highly secreted by glioma cells. Previous studies have shown that overexpression of PAI-1 in GBM is significantly correlated with shorter survival [11] and, that PAI-1 serum level could be attributed as a predictive marker for glioma grade [12]. Using microarray expression data and a larger cohort of 518 patients from the TCGA, we found that there is a statistically significant difference in survival between patients with low versus high PAI-1 levels (Figure 1A). Therefore, we decided to study the chemotactic capacity of PAI-1 by attempting to block MC chemotaxis by inhibiting PAI-1. As shown in Figure 2A, neutralization of the PAI-1 in U2987MG-conditioned medium with antibodies significantly lowered the motility of LAD2 MCs. A reduction of migration was even more pronounced when both MIF and PAI-1 in the conditioned medium were blocked (Supplementary Figure 1). Furthermore, the levels of PAI-1 in patient-derived glioma cell supernatants as compared to U2987MG were verified by using ELISA in both Proneural (U3016MG, U3047MG, U3117MG) and Mesenchymal (U3065MG, U3060MG, U3062MG, U3101MG) subtypes with previously identified low (U3016MG, U3047MG, U3065MG) and high (U3117MG, U3101MG, U3060MG, U3062MG) mRNA level of PAI-1 (Figure 2B). We further extended our study of LAD2 cell migration using high and low PAI-1 expressing patient-derived glioma cell lines from the Proneural and Mesenchymal subtypes. A direct correlation between subtype of glioma cells, level of PAI-1 and migration of LAD2 cells was observed, being highest in Mesenchymal subtype with high level of PAI-1 expression and lowest in Proneural subtype with low level of PAI-1 expression (Figure 2C).

### The extent of MC recruitment is correlated with the level of PAI-1 in human high-grade glioma

We have previously shown that the number of MCs in human glioma is dependent on malignancy grade. We identified PAI-1 as a highly expressed glioma-derived candidate [9], whose elevated expression in GBM has been demonstrated to be associated with short survival [11]. In addition, our *in vitro* data demonstrated a chemoattractant role of PAI-1 towards MCs. Therefore we proceeded with our studies by performing tissue analysis of the number of infiltrating MCs and SERPINE1 expression in human high-grade glioma TMAs.

**Figure 3:**
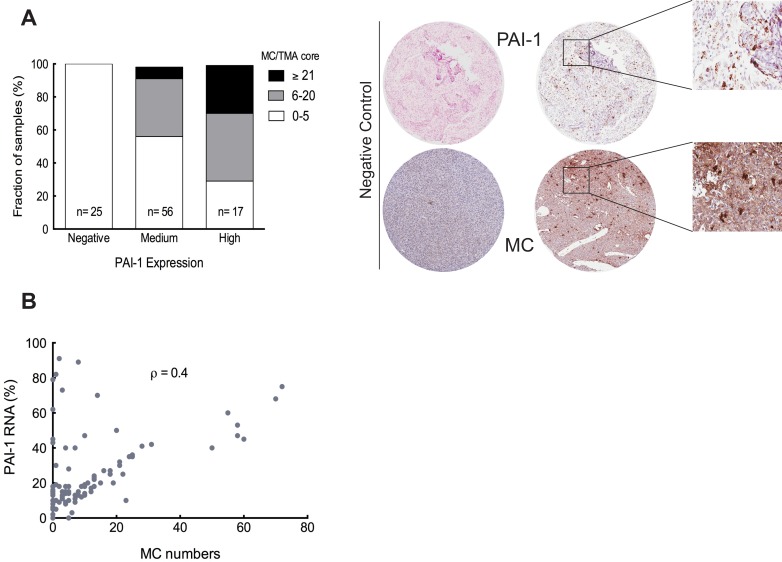
The level of PAI-1 is correlated with the extent of MC recruitment (**A**) Left panel: The relationship between the number of MCs and the PAI-1 expression level, specifically the fraction of PAI-1 mRNA expression per TMA core, ** *p* < 0.01. Right panel: Representative TMA cores of negative (left panels) and positive staining (middle panels) for tryptase (IHC) and PAI-1 RNA (ISH). Selected areas from middle panels are magnified in the right panels. (**B**) Spearman's rank correlation analysis between MC numbers and PAI-1 mRNA expression level, ρ = 0.4

PAI-1 is a widely expressed protein in glioma tissues, which resulted in a strong and widespread cytoplasmic staining when immunohistochemistry was performed (data not shown). The extent of intense and diffused staining made it unsuitable for quantification of PAI-1 in the glioma TMAs. Therefore, to investigate the potential correlation between the populations of GBM cells expressing SERPINE1 and the presence of MCs, we used RNA-*in-situ* hybridization (RNA-ISH) on high-grade glioma TMA's. Analysis of consecutive sections of the TMAs revealed a correlation between the number of infiltrating MCs and the relative staining intensity for PAI-1 (Figure 3A). Thus negative staining was associated with low MC numbers (0-5 MCs per TMA core) in all cases (n = 25). The proportion of TMA cores with low numbers of MCs was 57% (n = 32) among those with medium PAI-1 expression. The proportion of MCs between medium MC numbers (6-20 MC/TMA core) and high (≥21 MC/TMA core) numbers in TMA cores with medium PAI-1 expression was calculated as 35% (n = 20) and 7% (n = 4) respectively. The proportion of TMA cores exhibiting low numbers of MCs was lowest with high PAI-1 expression. These values were 29% (n = 5) for low MC numbers, 41% (n = 7) with medium and 29% (n = 5) with high MC numbers of high PAI-1 expressing samples. Representative positive and negative staining for MCs and PAI-1 is illustrated in left panel of Figure 3A.

A Spearman's correlation analysis comparing the MC numbers and PAI-1 expression showed positive correlation between them (Figure 3B). So we can conclude that high PAI-1 expression in the glioma tissue is associated with MC infiltration.

### Identification of LRP1 expression in MCs in human glioma and LAD2 cells is associated with their recruitment towards glioma-derived PAI-1

MCs express a variety of both cell surface as well as transmembrane receptors. However, none of the receptors was identified to interact with PAI-1 as yet. PAI-1 can bind to various matrix components e.g., vitronectin and LRP1, leading to dramatic consequences on their migratory phenotype [14]. In addition, previous publications demonstrated that PAI-1 stimulates macrophage motility in a LRP1 dependent manner [15].

**Figure 4:**
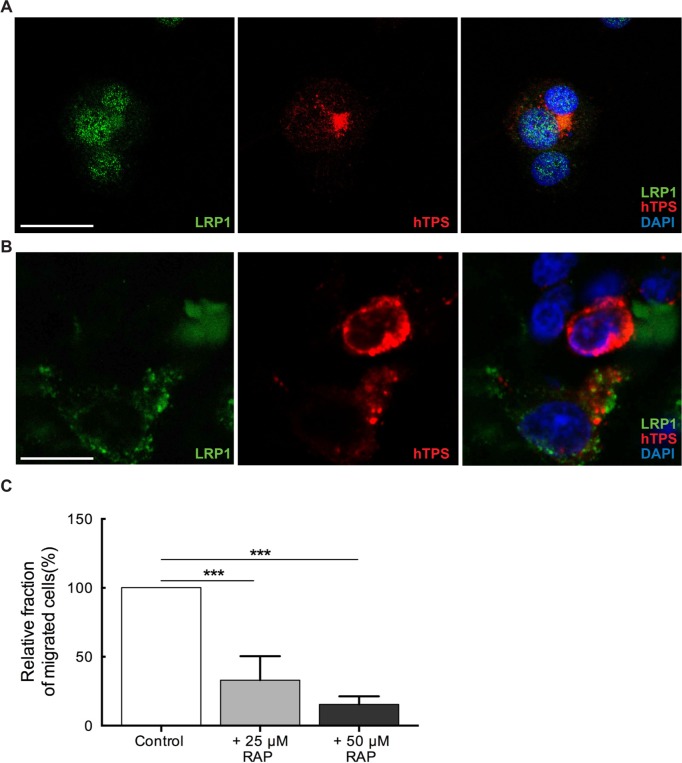
MCs constitutively express LRP1 (**A**) Immunofluorescence staining for LRP1 and human tryptase (hTPS) in LAD2 cells. Scale bar = 25 μM. (**B**) Immunofluorescence staining for LRP1 and human tryptase (hTPS) in human GBM tissue. Scale bar = 25 μM. (**C**) Blocking of LRP1 by receptor associated protein (RAP) attenuates the migration of MCs. LAD2 cells were treated with 25 μM and 50 μM RAP, 30 min prior to migration assay. U2987MG-conditioned medium was used as the chemoattractant. Three independent experiments were performed with triplicates. The error bars represent the SD. *** *p* < 0.001.

LRP1, one of the largest members of the LDLR family is synthesized as a 600 kDa precursor protein and processed in the trans-Golgi by a furin-like protease to yield a 515 kDa alpha-chain and an 85 kDa beta-chain that associates non-covalently [16]. The alpha chain contains four ligand-binding domains (clusters I-IV). We hypothesized that LRP1 is expressed on MCs and mediates MC motility towards glioma derived PAI-1. We identified LRP1 expression on LAD2 cells by observing the co-localization of LRP1 with human MC tryptase (hTPS) (Figure 4A). Similar staining was performed on human glioma tissue demonstrating the LRP1 expression on MCs *in vivo* (Figure 4B). To confirm the constitutive expression of LRP1 in MCs we stimulated LAD2 cells with PAI-1 enriched medium and then performed western blot (Supplementary Figure 2A) and RT-PCR (Supplementary Figure 2B) on LAD2 cells. The level of LRP1 expression was not altered and was consistent with or without stimulation by PAI-1, confirming that LAD2 cells express LRP1 constitutively.

To validate the importance of LRP1 in mediating MC's migratory capacity towards glioma-derived PAI-1, a low-density lipoprotein (LDL) receptor family blocker, receptor associated protein (RAP) was used to block LRP1 in LAD2 cells. RAP has been shown to bind with high affinity to cluster III of LRP1 [17]. The results demonstrated that migration of RAP pre-treated LAD2 cells towards PAI-1 enriched medium was significantly reduced in a dose-dependent manner (Figure 4C), being in line with our hypothesis that PAI-1 induces migration of LAD2 cells in a LRP1 dependent manner.

### Identification of direct interaction between PAI-1 and LRP1 in human glioma tissue by proximity ligation assay

**Figure 5:**
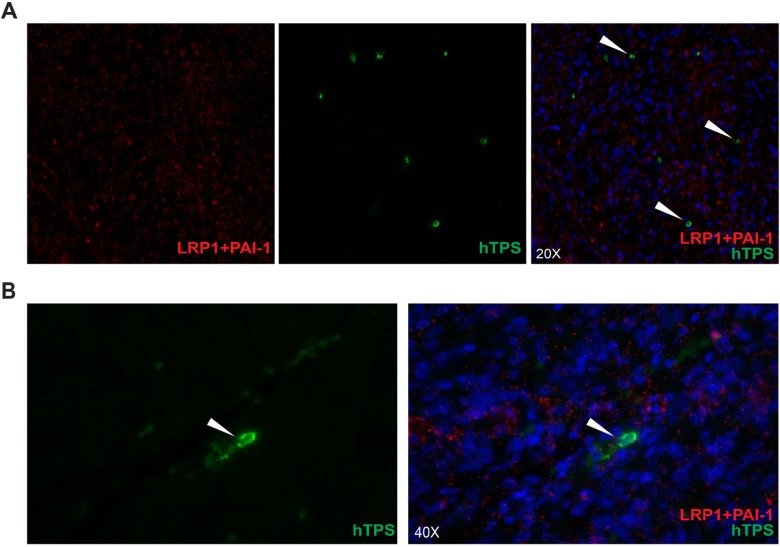
Identification of direct interaction between PAI-1 and LRP1 in human GBM tissue by proximity ligation assay The interaction between LRP1 on MCs and PAI-1 in vicinity in human GBM tissue (**A**) 20X, (**B**) 40X: Representative picture of proximity ligation assay in human GBM tissue showing the interaction between LRP1 and PAI-1 counterstained with human MC specific tryptase (hTPS) (also indicated by arrow heads).

According to the Human Protein Atlas (HPA) database, both PAI-1 and LRP1 are highly expressed proteins in glioma tissues (www.proteinatlas.org). Considering that LRP1 is expressed on other cell types in glioma in addition to MCs, we wanted to ascertain *in vivo* interaction of LRP1 and PAI-1in MCs. Proximity ligation assay (PLA) was used to detect binding of PAI-1 with LRP1 in glioma tissue. Positive co-localization signals (Figure 5A-5B) were abundant throughout the tumor tissue. To ascertain MC distribution/presence in the vicinity, the tissues were counter-stained with human MC specific protease, tryptase (hTPS) (Figure 5A-5B). We were able to detect that PAI-1 indeed binds to LRP1 in human glioma tissue attributing, to a certain extent, these interactions with LRP1 on MCs. Hence, these data elucidate an *in vivo* attractant role for PAI-1 leading to the observed high accumulation of MCs in glioma.

### PAI-1 promotes phosphorylation of STAT3 and regulation of exocytosis in MCs

**Figure 6:**
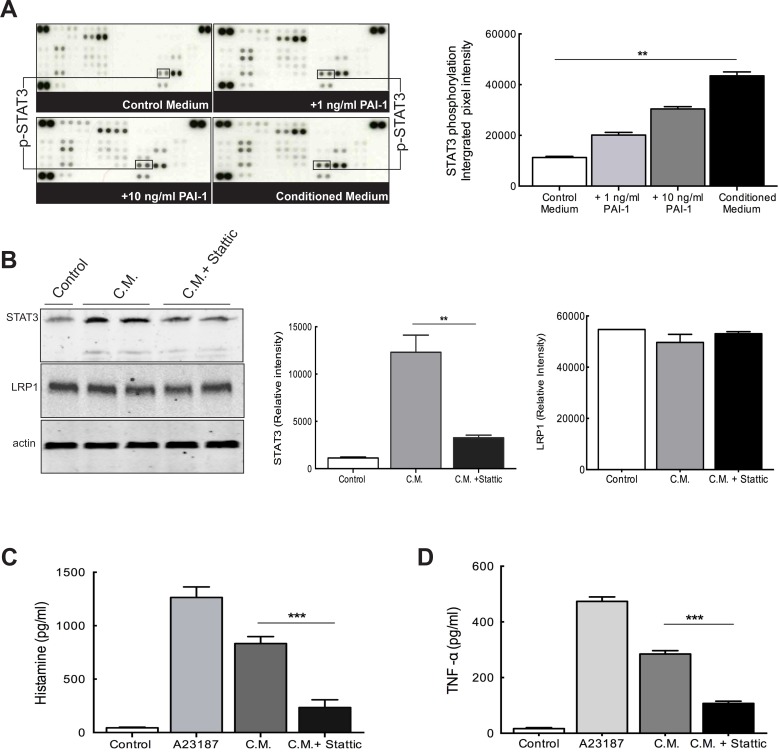
PAI-1 alters signaling networks in MCs (**A**) Upper panels: the phosphorylation profiles of lysates of MCs cultured in control medium (top left), medium containing PAI-1 (1ng/ml, top right; 10ng/ml, bottom left) or U2987MG-conditioned medium (C.M., bottom right). In the graph quantification of the dots of interest is plotted as integrated pixel intensities. The error bars represent the SD. ** *p* < 0.01. (**B**) Stattic can effectively inhibit STAT3 phosphorylation without changing LRP1 expression levels. Left panel: representative picture of a western blotting of LAD2 cells showing inhibition of STAT3 phosphorylation after treatment with Stattic, and equal LRP1 expression. Middle panel: quantification of western blotting to evaluate STAT3 levels. Right panel: quantification of western blotting to evaluate LRP1 levels. The error bars represent the SD. *** *p* < 0.001. Histamine levels (**C**) and TNF-alpha secretion (**D**) were measured by using ELISA and demonstrated that inhibition of STAT3 phosphorylation by Stattic reduces MC exocytosis. Three independent experiments were performed with triplicates. The error bars represent the SD. *** *p* < 0.001.

A phosphokinase array was used to identify possible targets downstream of signaling pathways induced by PAI-1. Protein lysates from PAI-1 stimulated LAD2 cells were analyzed by this array and revealed intense augmentation of the intracellular level of phosphorylated STAT3 (pSTAT3) as compared to control medium (Figure 6A).

STAT3 has been shown to be involved in MC functions by induction of various regulatory genes and its phosphorylation linked to MC exocytosis [18]. To test the potential role of STAT3 in MC exocytosis, Stattic, a nonpeptidic small molecule STAT3 inhibitor, was used [19]. LAD2 cells were pretreated with Stattic for 2 hours prior to stimulation by glioma-derived conditioned medium. The calcium ionophore A23187 was used as a positive stimulant of LAD2 cells. Western blotting verified the significant blocking of STAT3 phosphorylation by Stattic (Figure 6B). We also found that the level of LRP1 in the LAD2 cells remained unaltered (Figure 6B) after treatment with Stattic. This result further supports the constitutive expression of LRP1 in LAD2 cells demonstrated in Supplementary Figure 2.

The analysis of histamine release (Figure 6C) and TNF-alpha secretion (Figure 6D) by LAD2 cells after Stattic treatment demonstrated a significant decrease in both assays indicating an inhibition of MC exocytosis when STAT3 activation is hampered. Hence it is evident that PAI-1 stimulates active migration of MCs which, in turn, activates signaling cascades leading to STAT3 phosphorylation and, subsequently, causes MC degranulation in the tissue, thereby may trigger infiltration of other immune cells, development of angiogenesis and other modulation of the tumor microenvironment.

## DISCUSSION

The dependence of cancer growth on the reciprocal interactions between tumor cells and tumor microenvironment is increasingly recognized. Glioma microenvironment accommodates a diversity of cell types and a plethora of complex interactions between them. Inflammatory cells have been connected to tumor development by mediating the inflammatory response, which is critical for tumor formation.

Gliomas are infiltrated by a variety of immune cells from both the innate and adaptive immune systems. However, the correlation between these immune infiltrates and clinical outcome is controversial, leaving their role debatable and requiring more efforts in further investigations [20]. Despite the fact that the prevailing role for glioma-associated immune cells remains protumorigenic, it cannot be excluded that the functional plasticity of immune cells is dependent on multiple microenvironmental cues. These cues for example can be generated by immune cells and depend on the severity of glioma.

In our previous studies we demonstrated recruitment of MCs upon glioma development [8]. We also identified several glioma-secreted factors, which appeared to be crucial for MC recruitment [9]. One of the candidates identified was PAI-1. Considering previously shown direct correlation between overexpressed PAI-1 and the survival in GBM [11], and also its role in being potential serum marker in the prediction of glioma grade [12], we decided to focus our research on PAI-1.

Previous studies of the plasminogen-plasmin system in cancer suggested the importance of this system in facilitating tumor cell invasion by uPA-mediated activation of plasmin and subsequent degradation of extracellular matrix [21]. PAI-1 is the unique and specific inhibitor of both tPA and uPA [22] and thus expected to prevent plasmin generation. However, recent studies have brought forward another set of functions that are distinct from original function of uPA/PAI-1. These observations revealed that high PAI-1 expression is an indicator of poor prognosis in many cancers, suggesting the ambiguity of definition of PAI-1 as tumoricidal [10]. It was also demonstrated that there are nonproteolytic properties of PAI-1, including interactions with integrins, inhibition of apoptosis and promotion of tumor cell proliferation. It indicates the importance of other factors, including PAI-1 concentration, location, type of tumor, and presence of receptors, and not only the impact of PAI-1 induced proteolytic actions of plasmin.

The analysis of the TCGA GBM dataset revealed significant upregulation of SERPINE1 (PAI-1). Kaplan-Meier survival curves using the TCGA GBM dataset showed a significant difference in survival between patients with high versus low SERPINE1 expression. Further investigation of subtype-specific expression of SERPINE1 showed that the predominant subtype among the patients with highest SERPINE1 expression levels is the Mesenchymal subtype, while the Proneural subtype is predominant among patients with lowest SERPINE1 levels. Moreover, the survival analysis between the top/bottom 25% of patients within the Mesenchymal and Proneural subtypes showed that patients of Proneural subtype with low SERPINE1 levels have a statistically significant increase in survival compared with the other three groups. Based on these observations we hypothesized that PAI-1 expression could initiate MC migration in a subtype specific manner and eventually affect the role of MC in glioma development. To test this hypothesis we used the panel of patient-derived glioblastoma cell lines from the HGCC biobank with transcriptome data and assigned subtypes. The expression of SERPINE1 in this sample set mirrors the expression in the TCGA dataset, being highest in cell cultures of Mesenchymal subtype and lowest in the cell cultures of Proneural subtype. This demonstrated that given cell cultures are suitable models for studies of SERPINE1-related mechanisms in GBM biology.

In this study we identified PAI-1 as an important attractant of MCs in glioma. Moreover, on the basis of the TCGA and HGCC datasets we undertook a functional screen of the panel of patient-derived glioma primary cell cultures and confirmed the functional connection between glioma subtype dependent PAI-1 expression and MC migratory properties. LRP1 is known for its multiple functions in endocytosis and in signal transduction that allows it to activate inflammatory signaling pathways. Previous publications showed that LRP1 is capable of stimulating cytokine production by inflammatory cells and induce cell migration [23]. We are first to identify the LRP1 expression in MCs in both LAD2 cells and human gliomas and show that LRP1 was critical for accumulation of MCs towards PAI-1 expressing glioma cells. The neutralization of PAI-1 activation significantly attenuated MC migration. However, residual chemoattractant activity toward MCs was present even after adding the PAI-1 blocking antibody, suggesting the role for other MC chemoattractants. Indeed, MC migration was hindered even more noticeably when both MIF and PAI-1 blocking antibodies were added, suggesting a critical role for these chemoattractants in MC migration. Regulation of MC recruitment to gliomas by PAI-1 is supported further by our TMA data demonstrating a positive correlation between the level of PAI-1 mRNA expression and the number of intratumoral MCs.

At the same time PAI-1 promoted differential phosphorylation of a number of signaling molecules in MCs, including substantial enhancement of STAT3 phosphorylation and, subsequently, resulting in increased MC exocytosis. STAT3 plays a crucial role in tumor microenvironment. It propagates several levels of crosstalk between tumor cells and their immunological microenvironment. The activated STAT3 stimulates tumor stromal immune cells, whose recruitment promotes tumor progression [24, 25]. Our data is in line with the recent publications demonstrating the role for activated STAT3 in immunologically mediated MC exocytosis [18].

In conclusion, we demonstrated that glioma-derived PAI-1, inducing recruitment of MCs also mediates MC degranulation, releasing numerous MC mediators, which participate in glioma fate. These findings shed new light on the molecular basis of the interplay between glioma cells and MCs and eventually emphasize the impact of MCs upon glioma progression.

## MATERIALS AND METHODS

### Cell cultures

All cells were cultured at 37° C under 5% CO2. U2987MG, a human glioma cell line [26], was cultured in 10% FBS-containing MEM supplemented with 4mM L-glutamine, 100units/ml penicillin and 0.1mg/ml streptomycin. The U2987MG cell line was established from a patient with high-grade glioma [26] and was denoted as primary culture 18. This cell line have then been very well studied [27, 28] and subsequently established as a stable glioma cell line. U3016MG, U3047MG, U3117MG, U3065MG, U3060MG, U3062MG and U3101MG cells were cultured on laminin (10mg/ml) in serum-free (stem cell) conditions, to enrich for stem-like glioma cells, as described previously [29].

The human MC line LAD2 (obtained from Prof Dean Metcalfe at NIH/NIAID, MD, USA) was cultured as described previously [30] in StemPro medium supplemented with 4mM L-glutamine, 100units/ml penicillin and 0.1mg/ml streptomycin and 100ng/ml SCF (Invitrogen, Carlsbad, USA).

### Transwell migration assays

All migration experiments were carried out in a 24-well culture plates using transwells with 5μm PET membrane (Millipore, Billerica, MA). To evaluate the capacity of glioma cells to recruit MCs, conditioned media from confluent U2987MG, U3016MG, U3047MG, U3117MG, U3065MG, U3060MG, U3062MG and U3101MG cells were used as the chemoattractant with unconditioned media serving as controls. LAD2 cells were starved of SCF overnight, resuspended in control medium (5 × 10^4^ cells/ml), then allowed to migrate through transwells overnight and thereafter counted. Migration of MCs toward U2987MG-conditioned medium was set to 100%. These experiments were performed in triplicates and repeated at least 3 times.

Neutralizing experiments were performed in triplicates as described previously [8] with PAI-1 antibody concentration of 50μg/ml (R&D Systems, Abingdon, UK) and MIF antibody concentration of 50μg/ml (R&D Systems, Abingdon, UK). Control samples were incubated under same conditions with a matching isotype nonspecific IgG antibody (50 μg/ml; R&D Systems, Abingdon, UK). The selection of concentration range for PAI-1 neutralizing antibody was based on suggestion by manufacturers as determined by its ability to neutralize recombinant human PAI-1.

For blocking of LRP1 activity, cells were pretreated with two different concentrations (25μM and 50μM) of LRP1 antagonist RAP (Abcam, Cambridge, UK) for 30 min prior to migration studies.

### Inhibition and stimulation assays

To examine the effect of STAT3 on MC exocytosis, LAD2 cells were pre-treated with Stattic (Sigma Aldrich, St Loius, USA) an inhibitor of STAT3 phosphorylation, at a concentration of 80μM for 30 min at 37°C. The concentration of Stattic used was chosen based on previous publications [18] and published kinetic study experiments [19, 31]. Following this the LAD2 cells were stimulated with U2987MG media in a 24-well culture plate for 4 hours. Stimulation experiment was done in duplicates. Supernatants were collected for histamine and TNF-α ELISA. The calcium ionophore A23187 (Sigma Aldrich, St Loius, USA) was used as a positive control for stimulation. Supernatant from cells that were not treated neither with U2987MG medium or Stattic was used as unstimulated control.

### Western blot

Efficacy of Stattic (STAT3 inhibitor) inhibition on LAD2 cells as well as presence of LRP1 on LAD2 cells was confirmed using western blotting, as described elsewhere [29]. The primary antibodies used were directed against pSTAT3 (1:1000, Cell Signalling Technology, Danvers, MA), LRP1 (1: 5000, Abcam, Cambridge, UK) and β-actin (1:1000, Sigma Aldrich, St Loius, USA).

### RNA extraction, cDNA synthesis and RT-PCR

RNA was extracted from control LAD2 cells grown in different medium as well as from LAD2 cells after stimulation. RNA extraction was done using TRIzol® (Life Technologies, Waltham, MA) extraction method from cell pellets. cDNA was synthesized using the SuperScript® II Reverse Transcriptase (Invitrogen, Carlsbad, USA), which was then used to perform the PCR using the KAPA2G Fast PCR Kit (Kapabiosystems, MA, USA) to demonstrate LRP1 in LAD2 cells. The primers used for LRP1 in PCR are as follows: 5′-ACATATAGCCTCCATCCTAATC-3′ and 5′-TTCCAATCTCCACGTTCAT-3′.

### Phospho-kinase array

The human phospho-kinase array kit that allows for simultaneous detection of the changes in relative levels of phosphorylation of 46 kinase phosphorylation sites (R&D Systems; Abingdon, UK) was used in accordance with the manufacturer's directions. In brief, LAD2 cells grown overnight in the absence of serum were placed for 10-15 min in medium from confluent 96-h cultures of U2987MG cells or control medium with or without PAI-1 (1ng/ml and 10ng/ml). Lysates were prepared and applied to membranes overnight, after which signals were detected as described above after appropriate application of antibody cocktails and streptavidin-HRP solutions.

Quantification and analysis of the data was done using the ImageJ (NIH) software. Since capture antibodies are spotted onto the nitrocellulose membrane in duplicates, for quantification of both arrays, the pixel intensity of each spot was calculated, integrated and corrected for back- ground and the resulting average values from duplicate spots plotted. Integrated pixel intensity was determined for each spot, corrected for background and the resulting average values from duplicate spots plotted.

### *In vitro* measurement of histamine release and TNF-α secretion

Following stimulation of LAD2 cells with U2987MG-conditioned medium, the histamine levels (Oxford Biomedical Research, MI, USA) and TNF-α levels (R&D Systems, Abingdon, UK) in the media was measured by sandwich ELISA following the manufacturer's protocol. Both ELISA were done in triplicates for each stimulation assay.

### PAI-1 ELISA

PAI-1 levels in glioma cell supernatants and medium controls were measured by ELISA according to manufacturer's guidelines (Human PAI-1 Quantikine ELISA, R&D Systems, Abingdon, UK) on three replicates of 96 hours conditioned medium.

### Human tissue samples and tissue microarrays

Informed consent for the use of human brain tissue and for access to medical records for research purposes was obtained, and all material obtained in compliance with the Declaration of Helsinki. The experiments involving human tissue samples were approved both by the Ethics Committee of Uppsala University (Application Dnr Ups 02-330 and Ups 06-084) and the Ethics Committee of Karolinska Institutet (Application Dnr Ki 02-254) and written informed consent was solicited prior to collection of these samples. Human tissue samples were obtained from Uppsala Biobank material. High-grade gliomas had been graded based on the WHO classification by experienced neuropathologist.

Tissue microarrays (TMAs) and slide scanning were performed using the strategies of The Human Protein Atlas project (www.proteinatlas.org) [32, 33]. TMAs involved tumor tissue from high-grade gliomas (anaplastic gliomas and glioblastomas, n = 101).

### *In situ* hybridization

To identify the possible correlation between PAI-1 expression in high-grade gliomas and MC numbers, *in situ* hybridization (ISH) was performed to visualize single RNA molecules (PAI-1) per cell in glioma tissue samples. ISH was performed on high-grade glioma TMAs using RNAscope technology (Advanced Cell Diagnostics, Hayward, CA). The protocol described with the RNAscope® 2.0 High Definition (HD)-BROWN Assay kit was followed strictly throughout the experiment. Briefly, the FFPE TMA sections were deparaffinized and pretreated with citrate buffer and protease as instructed by the manufacturers. The slides were then hybridized sequentially with target probes, pre-amplifiers and amplifiers. The detection was done by the kits enhanced intense Diaminobenzidine (DAB) staining system followed by counterstaining with hematoxylin. The slides were mounted permanently.

A Hs-SERPINE1 probe (ACD-555961) was used to demonstrate expression of SERPINE1 RNA molecules in the glioma TMAs. Appropriate negative control probes was used as background control. Stained slides were scanned using the methods of The Human Protein Atlas project at ×40 magnification (www.proteinatlas.org) [32, 33]. Quantification of the RNA expression was done using the ImageJ software (NIH).

### Immunohistochemistry and immunofluorescence staining

Formalin-fixed, paraffin-embedded 6 μm thick tissue sections were fixed. Thereafter, the sections were deparaffinized (in xylene overnight, in fresh xylene for 1 h on a rocking table followed by 2 × 5 min incubations in 100% EtOH, 95% EtOH, 80% EtOH, distilled H2O) and subjected to pressure boiling for antigen retrieval in antigen unmasking solution (Vector Labs, Burlingame, CA).

Immunohistochemistry was performed using the UltraVision LP detection System (Thermo Fisher Scientific, Waltham, MA) in accordance with the manufacturer's instructions. Briefly, after antigen retrieval the slides were washed in PBS-T (containing 0.05% Tween (Sigma Aldrich, St Loius, USA) and incubated with hydrogen peroxidase block. Ultra V block was subsequently applied. Primary antibody used was anti-human tryptase (hTPS) (1:200, Santa Cruz Biotechnology, Santa-Cruz, USA) diluted in 5% milk in PBS-T. Primary antibody was applied overnight at 4°C, followed by primary antibody enhancer. Slides were incubated with HRP polymer and the signal was visualized using freshly prepared DAB plus chromogen and substrate mix. Between all the steps described above, the slides were thoroughly washed in PBS-T. After the final step, the slides were washed in distilled H2O, counterstained with hematoxylin and mounted using Immu-mount (Thermo Fisher Scientific, Waltham, MA). The slides were scanned using the methods of The Human Protein Atlas project (www.proteinatlas.org).

For immunofluorescence staining, slides were rinsed in PBS, blocked in 5% milk-containing PBS-Tx (supplemented with 0.2% Triton-X 100 (Sigma Aldrich, St Loius, USA)) for 1 hour, followed by overnight incubation (4°C) with the primary antibody diluted in the blocking solution. The following antibodies with the specific dilutions were used: LRP1 (1:250, Abcam, Cambridge, UK), PAI-1 (1:200 Santa Cruz Biotechnology, Santa-Cruz, USA), hTPS (1:250, Santa Cruz Biotechnology, Santa-Cruz, USA). The slides were subsequently incubated with appropriate secondary antibody for 45 min. Nuclei was stained with DAPI (1:5000) for 15 min and mounted in Immu-mount. All secondary antibodies used were Alexa antibodies (Invitrogen, Carlsbad, USA). Pictures were taken using Zeiss 510 META confocal microscope and Zen software (version 5.0, 2008). Where applicable, maximum intensity projection was performed on z-stack images.

### Proximity ligation assay

Proximity ligation assay (PLA) was done using the Duolink^®^ In Situ Red Starter Kit Mouse/Rabbit kit (DUO92101, from Duolink Sigma, St. Louis, USA), following the manufacturers instructions. Antibodies identifying PAI-1 and LRP1 of human origin were used. Briefly, mouse monoclonal PAI-1 (Santa Cruz Biotechnology, Santa-Cruz, USA) and rabbit polyclonal anti-LRP1 antibody (Abcam, Cambridge, UK) were tagged with anti-mouse MINUS and anti-rabbit PLUS probes and applied on the tissue. Following several washing and ligation steps as per the kit instructions, the signal was visualized using signal amplification of red fluorescent detection agent. The tissue was then mounted with DAPI and the coverslip sealed. The signal was visualized and images taken as Z-stack with ZEISS AxioImager microscope for fluorescence and ZEN Blue software.

### Bioinformatic analysis

Survival analysis was done using the TCGA GBM data downloaded from the cBio and TCGA portals. SERPINE1 TCGA expression data (TCGA provisional, Affymetrix GeneChip Human Genome U133 Plus 2.0 Array, for 528 patients) [34] was downloaded from the cBio Portal (http://www.cbioportal.org/). Clinical data for the TCGA patients was obtained from the TCGA portal and in each case the maximum value of the survival times reported for a patient was used. Transcriptome data for the HGCC collection was generated on Affymetrix GeneChip Human Exon 1.0 ST Arrays, RMA normalized and transformed in *z*-scores. HGCC cell lines were classified based on their expression profile for known signature genes [13] using known TCGA subtypes as reference and a *k*-nearest neighbor algorithm as described elsewhere (Xie et al. 2015, manuscript submitted). All data manipulations were done in R [35].

### Statistical analysis

Statistical analyses were done using the Graphpad Prism software (GraphPad Sowtware 6.0d). For group-wise comparisons the Student's unpaired *t*-test was used. For comparisons between more than two groups ANOVA was applied. Kaplan-Meier survival analysis was used for the TCGA and HGCC data and was performed with censoring at confidence interval 95%. The *p* values for the survival analysis are for the log-rank test.

## SUPPLEMENTARY MATERIAL FIGURES


