# Supplementary material for: Glioma-derived plasminogen activator inhibitor-1 (PAI-1) regulates the recruitment of LRP1 positive mast cells
Source: Oncotarget. 2015 Jun 25;6(27):23647–61. doi: 10.18632/oncotarget.4640 (PMC4695142; doi:10.18632/oncotarget.4640)
Supplement: Supplementary file 1 [file oncotarget-06-23647-s001.pdf]

# Glioma-derived plasminogen activator inhibitor-1 (PAI-1) regulates the recruitment of LRP1 positive mast cells

**Supplementary Material**

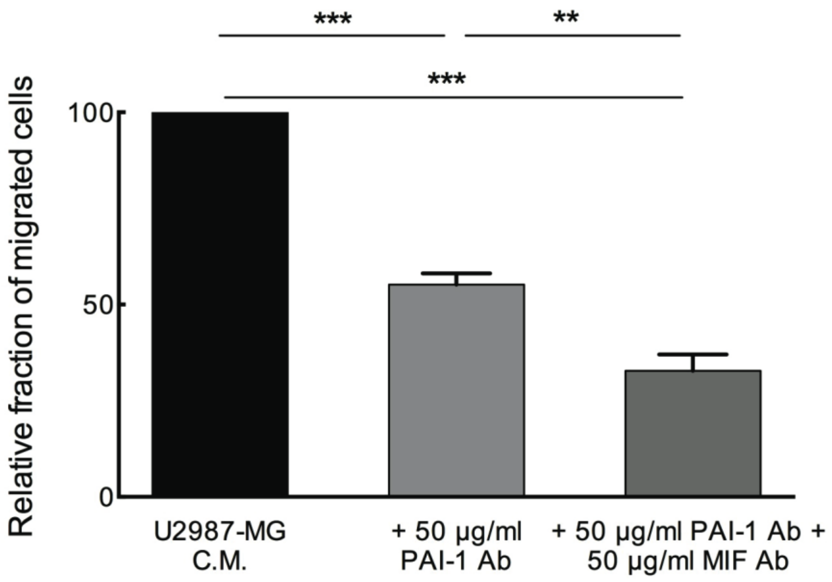


**Supplementary Figure 1**. **Neutralization of glioma-derived PAI-1 and MIF significantly attenuates the migration of MCs toward glioma cell-conditioned medium.** Migration of MCs towards i) conditioned media (C.M.) from U2987MG glioma cell line, ii) C.M. neutralized with 50µg/ml PAI-1 antibody, iii) C.M. neutralized with 50µg/ml PAI-1 antibody and 50µg/ml MIF antibody and iv) C.M. incubated under the same conditions with a matching isotype nonspecific IgG antibody (50 µg/ml). The experiments were performed 3 times, with duplicates in each case. The error bars represent the SD. *** $p < 0.001$, ** $p < 0.01$.

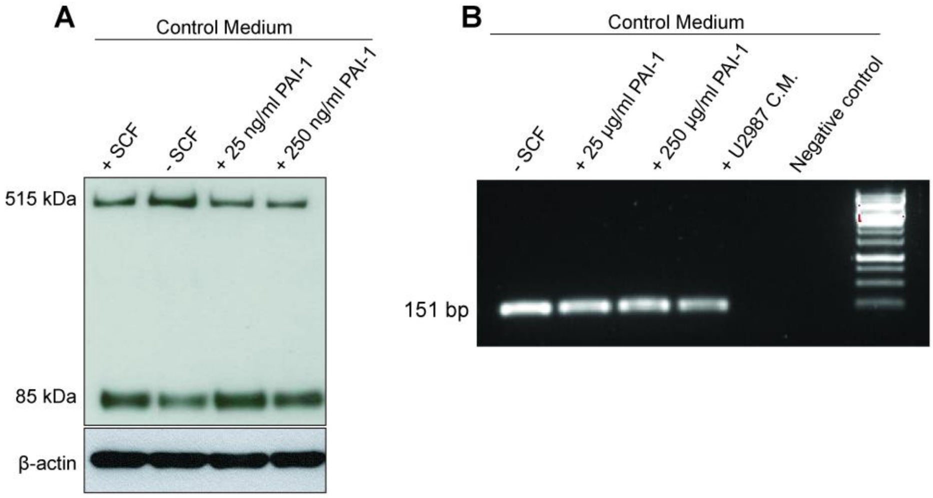


**Supplementary Figure 2**. **MCs constitutively express LRP1.** (**A**) LAD2 cells were grown in their normal culture medium (-SCF and +SCF) as well as under different conditions (25ng/ml PAI-1 and 250ng/ml PAI-1) and U2987MG-conditioned medium (C.M.). Western blot was performed to check for LRP1 expression in the cells. (**B**) LAD2 cells were grown in their normal culture medium (-SCF) as well as under different conditions (25ng/ml PAI-1 and 250ng/ml PAI-1) and U2987MG-conditioned medium. Cells were lysed and RNA was purified. PCR was done to check for the presence of LRP1 in the cells.
